# Maternal cardiometabolic dysfunction and fetal sex‐specific alterations to uterine vascular reactivity in an ovine model of obesity during pregnancy

**DOI:** 10.1113/JP290766

**Published:** 2026-05-22

**Authors:** Rachael C. Crew, Anna L. K. Cochrane, Youguo Niu, Sage G. Ford, Clement L. R. Cahen, Skaai H. Davison, Michael P. Murphy, Susan E. Ozanne, Dino A. Giussani

**Affiliations:** ^1^ Department of Physiology, Development and Neuroscience University of Cambridge Cambridge UK; ^2^ Department of Obstetrics and Gynaecology University of Cambridge Cambridge UK; ^3^ School of Human Sciences University of Western Australia Perth Western Australia Australia; ^4^ Loke Centre for Trophoblast Research University of Cambridge Cambridge UK; ^5^ Strategic Research Initiative in Reproduction University of Cambridge Cambridge UK; ^6^ Cardiovascular Strategic Research Initiative University of Cambridge Cambridge UK; ^7^ MRC Mitochondrial Biology Unit, Department of Medicine University of Cambridge Cambridge UK; ^8^ Department of Medicine University of Cambridge Cambridge UK; ^9^ Institute of Metabolic Science, Metabolic Research Laboratories and MRC Metabolic Diseases Unit University of Cambridge Cambridge UK

**Keywords:** cardiovascular, fetus, maternal, obesity, placenta, pregnancy, uterine artery

## Abstract

**Abstract:**

Obesity during pregnancy is at pandemic proportions and predisposes women to pre‐ and postnatal cardiovascular dysfunction. The mechanisms underlying this maternal cardiovascular vulnerability remain unclear, partly due to a lack of translatable models capable of longitudinal *in vivo* cardiovascular monitoring. Here, we characterize a novel ovine model of maternal diet‐induced obesity during pregnancy. Ewes were fed a control (CON) or obesogenic (OB; *ad libitum* concentrates) diet for 60 days pre‐pregnancy and throughout gestation. Pregnant ewes were surgically instrumented with vascular catheters and Transonic flow probes using the wireless CamDAS system, which measured maternal cardiovascular function near term in free‐moving ewes. Uterine artery vasoreactivity was assessed *ex vivo* by *in vitro* wire myography. OB ewes entered pregnancy 30% heavier than controls (*P* < 0.003) and were hyperglycaemic, hyperinsulinaemic and hyperlipidaemic during pregnancy, relative to CON ewes (all *P* < 0.05). OB ewes had elevated haematocrit and haemoglobin across pregnancy, and were hypertensive near term, with an increase in basal femoral artery blood flow, and elevated peripheral oxygen and glucose delivery (all *P* < 0.05). OB mothers carrying a female fetus showed increased uterine artery vascular resistance *in vivo* (*P* < 0.005) and reduced smooth muscle‐dependent vasorelaxation *ex vivo* (*P* < 0.05) relative to CON. Conversely, OB mothers carrying a male fetus showed greater NO‐independent mechanisms mediating the uterine vasodilator response to methacholine *ex vivo* (*P* < 0.001). Collectively, this study characterizes a robust model of maternal obesity during pregnancy that offers clinical translational potential and highlights fetal sex‐specific changes to uterine artery function.

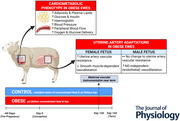

**Key points:**

- Obesity during pregnancy is increasingly common and predisposes women to cardiovascular dysfunction during pregnancy and long after birth, but the specific mechanisms underlying this remain unclear.
- We developed a novel ovine model of diet‐induced obesity during pregnancy that displays maternal hypertension, elevated haemoglobin, metabolic dysfunction, and alterations in uterine and peripheral blood flow and nutrient delivery near term.
- Mothers with obesity carrying a female fetus had elevated uterine vascular resistance *in vivo* and reduced uterine artery smooth muscle‐dependent vasodilator reactivity *ex vivo*.
- Mothers with obesity carrying a male fetus showed no effect on uterine vascular resistance *in vivo*, but greater NO‐independent mechanisms mediating the uterine vasodilator response to methacholine *ex vivo*.
- These findings highlight that fetal sex may influence maternal cardiovascular function during obese pregnancy.

## Graphical abstract

**Abstract figure legend** We developed a novel ovine model of diet‐induced maternal obesity, where ewes were fed a control or obesogenic diet for 60 days pre‐pregnancy and throughout gestation. Pregnant ewes were surgically instrumented to assess maternal cardiovascular function near term. Mothers with obesity displayed increased adiposity, metabolic dysfunction, elevated haemoglobin, hypertension, and alterations to peripheral blood flow and nutrient delivery. Maternal obesity also induced several fetal sex‐specific uterine artery adaptations. Obese ewes carrying a female fetus had elevated uterine vascular resistance *in vivo* and reduced uterine artery smooth muscle‐dependent vasodilator reactivity *ex vivo*, while those carrying a male fetus showed no effect to uterine vascular resistance *in vivo*, but greater NO‐independent mechanisms mediating the uterine vasodilator response to methacholine *ex vivo*. This highlights that fetal sex may influence maternal cardiovascular function during obese pregnancy.

## Introduction

Women continue to be underdiagnosed, undertreated and underrepresented in cardiovascular science, with research failing to sufficiently address factors that uniquely affect a woman's cardiovascular risk across the life course (Vervoort et al., 2024). Women who develop gestational complications are more susceptible to cardiovascular dysfunction during pregnancy, and this heightened cardiovascular vulnerability can continue long after delivery (Parikh et al., 2021; Täufer Cederlöf et al., 2022). Maternal obesity is a major risk factor for pregnancy complications, which is of the gravest concern given that rates of obesity in women of reproductive age have reached pandemic proportions (Kent et al., 2024; Schon et al., 2024). However, the mechanistic links between obesity during pregnancy and maternal cardiovascular dysfunction remain unclear, partly due to a lack of experiments in animal models of increased human translational potential that permit invasive longitudinal *in vivo* monitoring of maternal cardiometabolic function.

The ovine model offers distinct translational advantages in pregnancy research, thereby bridging the gap between human populations and mechanistic preclinical studies. Ovine studies add a new dimension to what can be addressed by the widely used rodent models of maternal obesity during pregnancy, which are used by many laboratories, including ours (for a review see Cochrane et al. 2024). In contrast to altricial, litter‐bearing rodents, sheep share similar developmental milestones to humans, exhibiting a relatively long gestational period, and giving birth to precocial singleton or twin offspring with comparable birth weights to humans (Morrison et al., 2018). The ovine placenta, while anatomically distinct, shares physiological similarities with humans, including counter‐current flow of maternal and fetal blood within the placental villous tree, almost identical oxygen gradients and consumption rates (37 *vs*. 34 mL/kg/min in sheep and humans, respectively), and similar glucose transfer mechanisms and nutrient transporter expression profiles (Barry & Anthony, 2008; Bonds et al., 1986; Ma et al., 2011; Regnault et al., 2013; Wilkening et al., 1988). These similarities in fetal size and nutritional demand induce comparable maternal cardiometabolic adaptations to pregnancy between sheep and humans. Moreover, surgical instrumentation of the maternal–fetal vasculature is possible in sheep, which enables serial blood sampling and longitudinal measurement of *in vivo* cardiometabolic function (Allison et al., 2016; Tong et al., 2022).

Here, we report the development and characterization of a novel ovine model of obesity during pregnancy that displays maternal metabolic dysfunction, hypertension, and alterations in uterine and peripheral blood flow and nutrient delivery near term. We further show fetal sex‐specific alterations to *in vivo* uterine artery function and *ex vivo* uterine artery reactivity in mothers with obesity, highlighting the impact of maternal–fetal communication on maternal vascular adaptations to pregnancy. This offers mechanistic insight into vascular disruption in a clinically accessible and routinely monitored vascular bed, thereby providing enhanced translational potential.

## Materials and methods

### Ethical approval

These studies were conducted in multiparous 2–3‐year‐old Welsh Mountain ewes at the Barcroft Centre of the University of Cambridge. All procedures involving animals were performed under the UK Animals (Scientific Procedures) Act 1986 (Project licences: PC6CEFE59/PP6755721), following approval by the University of Cambridge Animal Welfare and Ethical Review Board. All investigators adhered to the ethical principles and reporting standards for animal experiments outlined by Grundy (2015). The experimental design followed the recommendations of the ARRIVE (Kilkenny et al., 2012) and the National Centre for Replacement Refinement and Reduction (NC3Rs) guidelines (Tannenbaum & Bennett, 2015).

### Feeding regimen and pregnancy establishment

Following a minimum of 2 weeks of acclimatization to the facility, Welsh Mountain ewes were fed a control diet (CON, recommended ration of concentrates; 200 g/day per sheep, Bearts Ewe Nuts; H & C Beart Ltd, Norfolk, UK, and *ad libitum* hay), or an obesogenic diet (OB, *ad libitum* access to the same concentrates and hay) for at least 60 days pre‐pregnancy and throughout gestation. Body condition scores between 1 (emaciated) and 5 (obese) were obtained bi‐weekly by two of all trained assessors (RCC, SGF, YN, ALKC, CLRC) via palpation of the transverse and vertical processes of the lumbar vertebrae (Kenyon et al., 2014). Following 8–10 weeks of the feeding regimen, oestrus was synchronized in ewes via the insertion of a controlled release flugestone acetate vaginal sponge (Chronogest® CR 20 mg). Ewes were then housed with a stud ram for 5 days, with the date of raddle marking designated as day 0 of gestation. Pregnancy was confirmed by an ultrasound scan at 80 days of gestational age (dGA). Term in this breed is ca. 147 days (Brain et al., 2019). All ewes were maintained on their respective CON or OB diet throughout the experimental period.

### Longitudinal blood sampling protocol

Serial blood samples (10 mL) were taken from the external jugular vein in a subset of ewes at baseline (prior to CON or OB diet allocation), after 4 and 8 weeks of pre‐pregnancy diet exposure, at marking (peri‐conception), and at 40, 80 and 120 dGA. Blood samples were analysed for glucose, haemoglobin (Hb) and haematocrit (Hct) with an ABL90 FLEX PLUS blood gas analyser (Radiometer Ltd, Crawley, UK). The remaining EDTA‐prepared blood samples were centrifuged at 2370 x *g* for 5 min. Plasma aliquots were snap‐frozen in liquid nitrogen and stored at −80°C until further analyses.

### Maternal surgical instrumentation

At 117 ± 2 dGA, under general anaesthesia, a subset of pregnant ewes (*n* = 8 CON; 6 singleton, 2 twin, *n* = 12 OB; 8 singleton, 4 twin) were surgically instrumented with vascular catheters and perivascular flow probes to measure maternal cardiovascular function, as described previously (Allison et al., 2016, 2020; Tong et al., 2022). Briefly, ewes were fasted for 24 h prior to surgery with continuous access to water. On the day of surgery, anaesthesia was induced in animals with a jugular vein injection of Alfaxan (1.5–2.5 mg/kg alfaxalone; Jurox Ltd, UK). Ewes were then intubated (Portex cuffed endotracheal tube; Smiths Medical International Ltd, UK) using a laryngoscope for maintenance of general anaesthesia using 1.5–2.0% isoflurane (IsoFlo; Abbott Laboratories Ltd, UK) in 60:40 O_2_:N_2_O during spontaneous breathing. The maternal abdomen, flanks and medial surfaces of the hind limbs were then shaved and cleaned, and pre‐operative antibiotics (30 mg/kg procaine benzylpenicillin i.m.; Depocillin; Intervet UK Ltd, UK) and an analgesic agent (1.4 mg/kg carprofen s.c.; Rimadyl; Pfizer Ltd, UK) were administered. The ewe was then transferred to the surgical theatre and general anaesthesia was maintained using a positive pressure ventilator (Datex‐Ohmeda Ltd, UK). The animal was covered with sterile drapes, a midline abdominal incision was made and a Transonic flow probe (MC4PSB‐JS‐WX120‐CM4B‐GC; Transonic Systems Europe, Netherlands) was positioned around one of the main uterine arteries. Bilateral incisions were made within the maternal femoral triangles, and catheters were inserted into the right maternal femoral artery (Teflon; ID, 1.0 mm; OD, 1.6 mm; Altec, UK) and vein (polyvinyl chloride; ID, 0.86 mm; OD, 1.52 mm; Critchley Electrical Products, NSW, Australia) with the tips placed in the descending aorta and inferior vena cava, respectively. A second Transonic flow (MC4PSB‐JS‐WX120‐CM4B‐GC) probe was implanted around the left maternal femoral artery. All catheters and flow probe leads were exteriorized through keyhole incisions on the ewe's flanks. While under general anaesthesia, the ewe was fitted with a bespoke jacket housing the CamDAS, a wireless data acquisition system developed in our laboratory (Allison et al., 2016, 2020; Tong et al., 2022). The arterial catheter was connected to a pressure transducer within the pressure box unit, and the Transonic flow probes were connected to the flow box unit housed in the jacket (Allison et al., 2016, 2020; Tong et al., 2022). The dead space of each catheter was then filled with heparinized saline (100 IU/mL heparin in 0.9% NaCl). Isoflurane was withdrawn and the ewe was extubated after spontaneous breathing had returned.

During post‐operative recovery, ewes were housed in individual floor pens under a 12:12 h light:dark cycle and maintained on their respective CON or OB diets. Analgesia and antibiotic administration continued for 3–5 days following surgery, as described previously (Allison et al., 2020). All catheters were flushed daily with heparinized saline to maintain patency, and maternal arterial blood gas measurements were taken daily to monitor ewe wellbeing.

### Maternal cardiovascular recording and nutrient delivery calculations

After 5 days of post‐operative recovery (122 ± 2 dGA), descending aortic blood pressure (via the femoral artery catheter) and blood flow in the femoral and uterine arteries were recorded continuously on a beat‐to‐beat basis in each free‐moving ewe. Cardiovascular data were visualized on IDEEQ 2.15.0 software and analysed in LabChart 8 to calculate the systolic, diastolic and mean arterial blood pressure. Heart rate was calculated from the femoral artery flow pulse. Vascular resistance in the uterine and femoral arterial circulations was calculated using Ohm's principle, by dividing the mean arterial blood pressure by the respective blood flow. Blood gas samples were taken from the femoral artery catheter at the start of the cardiovascular recording period. The maternal arterial blood oxygen content ($C_{aO_{2}}$) was then calculated as:

$$C_{aO_{2}}\;\left(\mathrm{mmol}/L\right)=\mathrm{Hb}\;\times \;S_{O_{2}}/100\;\times \;0.62$$

where Hb is haemoglobin concentration (g/L), $S_{O_{2}}$ is oxygen saturation (%) and one molecule of Hb (MW 64.450) binds four molecules of oxygen, as described previously (Allison et al., 2016, 2020; Gardner et al., 2003). The contribution of oxygen dissolved in plasma was considered negligible (Owens et al., 1987).

Oxygen and glucose delivery to the maternal peripheral and uterine vascular beds was then calculated according to the following equations:

$$\begin{array}{ccc} & & \mathrm{oxygen}\;\mathrm{delivery}\left(\mathrm{mmol}/\min\right)=C_{aO_{2}}\;\left(\mathrm{mmol}/L\right)\\ & & \;\times \;\mathrm{appropriate}\;\mathrm{flow}\left(\mathrm{mL}/\min\right) \end{array}$$

$$\begin{array}{ccc} & & \mathrm{glucose}\;\mathrm{delivery}\left(\mathrm{mmol}/\min\right)\\ & & =\mathrm{blood}\;\mathrm{glucose}\left(\mathrm{mmol}/L\right)\times \mathrm{appropriate}\;\mathrm{flow}\left(\mathrm{mL}/\min\right) \end{array}$$

### Post‐mortem tissue collection

At 130 dGA, blood samples were collected from the maternal jugular vein in a subset of non‐instrumented animals (*n* = 18 CON, 18 OB). Maternal blood pH, partial pressure of oxygen ($P_{O_{2}}$) and carbon dioxide ($P_{CO_{2}}$), oxygen saturation ($S_{O_{2}}$), haemoglobin (Hb), glucose, lactate and electrolytes were measured with the ABL90 FLEX PLUS blood gas analyser (Radiometer Ltd, Crawley, UK). Ewes in the instrumented and non‐instrumented groups were then humanely killed by an overdose of sodium pentobarbitone (0.4 mL/kg i.v. Pentoject; Animal Ltd, York, UK). The fetus was exteriorized via Caesarean section and weighed. Fetal measurements were taken, including crown–rump length (CRL), biparietal diameter (BPD), abdominal circumference (AC) and femur length. Fetal body mass index (BMI) was calculated as fetal body weight (kg)/CRL (cm)^2^ and Ponderal Index (PI) was calculated as fetal body weight (g) × 100/CRL (cm)^3^. Placentomes were isolated and classified as A, B, C or D subtypes, according to Vatnick et al. (1991). Each placentome type was counted and weighed individually. Maternal and fetal organs were also isolated and weighed. For bilateral organs, the right maternal organ and both fetal organs were weighed. When bilateral fetal organs showed no statistical difference according to anatomical position, weights were presented as the mean of both organs.

### Uterine artery wire myography

Following post‐mortem at 130 dGA, uterine artery reactivity was determined in a subset of randomly selected ewes (*n* = 12 CON, 13 OB) by *in vitro* wire myography. A third‐order branch of the uterine artery was isolated, and ca. 2 mm vessel segments were threaded with stainless steel 40 µm diameter wire and secured in microvascular chambers (Wire Myograph System 610M; DMT, Aarhus, Denmark). The optimal diameter was determined for each vessel according to Delaey et al. (2002). Briefly, vessels were stretched to a diameter of 400 µm and equilibrated in Krebs buffer (118.5 mm NaCl, 25 mm NaHCO_3_, 4.7 mm KCl, 1.2 mm MgSO_4_.7H2O, 1.2 mm KH_2_PO_4_, 2.5 mm CaCl_2_, 2.8 mm d‐glucose; Sigma‐Aldrich, Gillingham, UK) bubbled with 95% O_2_/5% CO_2_ at 37°C. Once the tension recording was stable, the chamber was filled with high K^+^ Krebs (59.25 mm NaCl, 25 mm NaHCO_3_, 4.7 mm KCl, 1.2 mm MgSO_4_.7H_2_O, 64.86 mm KH_2_PO_4_, 2.5 mm CaCl_2_, 2.8 mm d‐glucose; Sigma‐Aldrich) and the maximum change in vessel tension recorded. Vessels were then washed in standard Krebs buffer and the vessel diameter increased by 25–100 µm increments, depending on the magnitude of response. When tension returned to baseline, the high K^+^ Krebs was again administered and the maximum change in tension recorded. These steps were repeated until the change in tension was equal to that reached at the previous vessel diameter, indicating that an optimal physiological working diameter was achieved (Delaey et al., 2002). Vessels were then washed with standard Krebs buffer and left to rest for at least 20 min before the generation of dose–response curves.

Uterine vascular constrictor function was determined by measuring tension to cumulative increasing doses of serotonin (5‐HT; 10^−9^ to 10^−4^ m). This 5‐HT dose–response curve was then repeated following a 20 min pre‐incubation with the synthetic Rho‐kinase inhibitor Y27632 to determine the Rho kinase‐dependent contribution to the vasoconstriction. The responses to the 5‐HT doses were normalized to the tension developed in response to maximal K^+^ (0 mm NaCl, 25 mm NaHCO_3_, 4.7 mm KCl, 1.2 mm MgSO_4_.7H_2_O, 125 mm KH_2_PO_4_, 2.5 mm CaCl_2_, 2.8 mm d‐glucose; Sigma‐Aldrich). Uterine vascular endothelium‐dependent dilator function was also determined by measuring tension changes to cumulative increasing doses of methacholine (10^−9^ to 10^−4^ m) following pre‐constriction with a sub‐maximal dose of 5‐HT. The methacholine dose–response curve was repeated following a 20 min pre‐incubation with nitric oxide (NO) synthase inhibitor l‐NAME (*NG*‐nitro‐l‐arginine methyl ester hydrochloride) to determine the contribution of NO‐dependent mechanisms to methacholine‐mediated vasodilatation, as previously established (Herrera et al., 2010). Uterine vascular smooth‐muscle‐dependent dilator function was determined by measuring tension changes to cumulative increasing doses of sodium nitroprusside (SNP; 10^−10^ to 10^−4^ m) following pre‐constriction with a sub‐maximal dose of 5‐HT. Vessel tension changes for determining constrictor or dilator reactivity in the uterine artery were recorded with LabChart software (LabChart 6.0, Powerlab 8/30; AD Instruments, Chalgrove, UK).

### Biochemical assays

Plasma insulin levels were quantified with an ovine‐specific insulin ELISA kit (Mercodia, Uppsala, Sweden). The intra‐assay coefficient of variation was 3%. Total plasma cholesterol (CHOL) and triglycerides (TG) were quantified by enzymatic assays, performed by the MRC MDU Mouse Biochemistry Laboratory (MC_UU_00014/5). Reagents were provided by Siemens Healthcare (Forchheim, Germany) and analysed on the Siemens Dimension EXL analyser. The limit of detection was 1.3 and 0.17 mmol/L for CHOL and TG, respectively.

### Statistical analyses

All data are presented as the mean ± SEM. Statistical analyses were performed with GraphPad Prism 9.5.1 software, with *P* < 0.05 considered statistically significant. Individual comparisons between CON and OB ewes were made by Student's *t* test for unpaired data. Diet and gestational age comparisons across longitudinal maternal samples were made using repeated measures two‐way ANOVA. When a significant (*P* < 0.05) interaction was present in ANOVA, the *post hoc* Šídák's comparison test was used to isolate significant relationships. Maternal diet, fetal sex and placentome type comparisons were made using three‐way or two‐way ANOVA, as appropriate. When no fetal sex effect was present (*P* > 0.05 in ANOVA), male and female data were combined. Placentomal data were further analysed by a two‐way mixed effects model, which allowed for nesting within each mother. In addition, all data were assessed for the impact of twinning and maternal instrumentation status via a generalized linear model (GLM) in R version 4.4.1. Groups were combined when no significant effect was present. For instance, fetal weights were included from both instrumented and non‐instrumented mothers. In cases of same‐sex twins, one twin was chosen at random to be included, to ensure that each pregnancy rather than fetus was the distinct biological replicate. Mixed sex‐twins were not included in this study. This resulted in data available from *n* = 13 CON male (11 singleton, 2 twin), *n* = 10 CON female (9 singleton, 1 twin), *n* = 11 OB male (10 singleton, 1 twin) and *n* = 10 OB female (9 singleton, 1 twin) fetuses.

## Results

### Ewe weight gain, body condition and dietary intake

**Figure 1:**
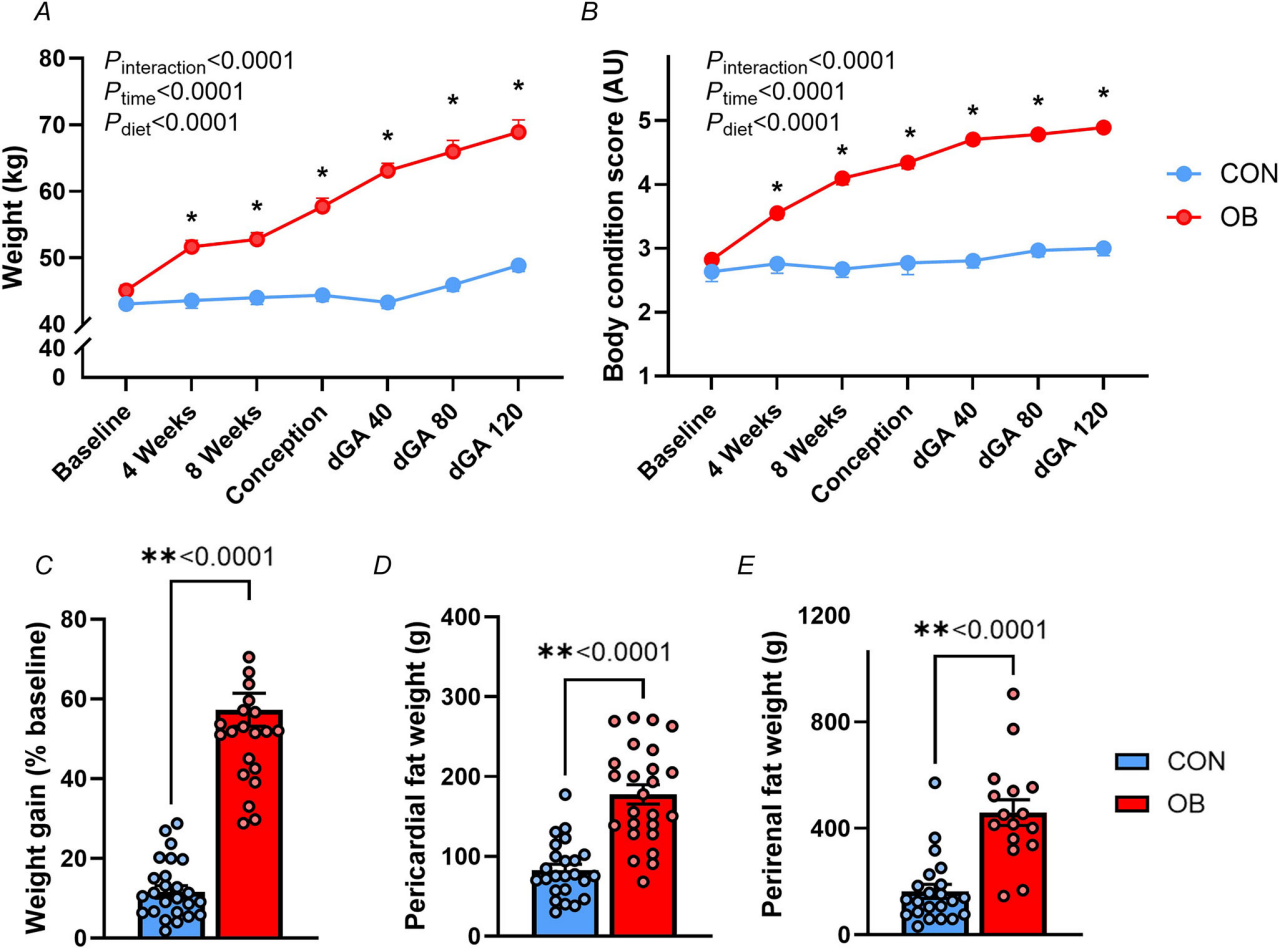
Ewe weight gain and adiposity *A*, ewe body weight; and *B*, condition score across the pre‐pregnancy, conception and pregnancy periods. *C*, total maternal weight gain by 120 dGA, expressed as a percentage of baseline weight; and *D*, maternal pericardial; and *E*, perirenal fat deposit weights upon post‐mortem at 130 dGA. All data are the mean ± SEM in control (CON; blue) and obese (OB, red) ewes, with *n = *18–41 ewes per diet group, per time point. *P < 0.003 CON *vs*. OB comparison; Šídák's multiple comparison test following *P* < 0.0001 Diet × Time interaction in two‐way repeated measures ANOVA. **P < 0.001; unpaired *t* test. AU; arbitrary units.

Analysis of food intake in a subset of ewes showed that the OB group (*n* = 21) consumed 967 ± 20 g of concentrated feed per day, compared to 200 ± 0 g in the CON group (*n* = 20). Therefore, OB relative to CON ewes had a ca. 5‐fold increase in daily energy intake. While there was no difference between groups in body weight or condition score at baseline, ewes in the OB group were significantly heavier and displayed an elevated body condition score after 4 weeks of diet exposure (Fig. 1*A* and *B*). This increased weight gain trajectory continued throughout the pre‐pregnancy feeding period, such that OB ewes entered pregnancy 30% heavier than CON ewes (Fig. 1*A*). OB ewes continued to gain more weight throughout pregnancy, such that their total percentage weight gain was 57% by 120 dGA, compared to 12% in CON ewes (Fig. 1*C*). By post‐mortem at 130 dGA, OB ewes had significantly greater adiposity, with a 115% increase in pericardial fat and a 183% increase in perirenal fat, relative to CON ewes (Fig. 1*D* and *E*).

### Longitudinal *in vivo* metabolic phenotype

**Figure 2:**
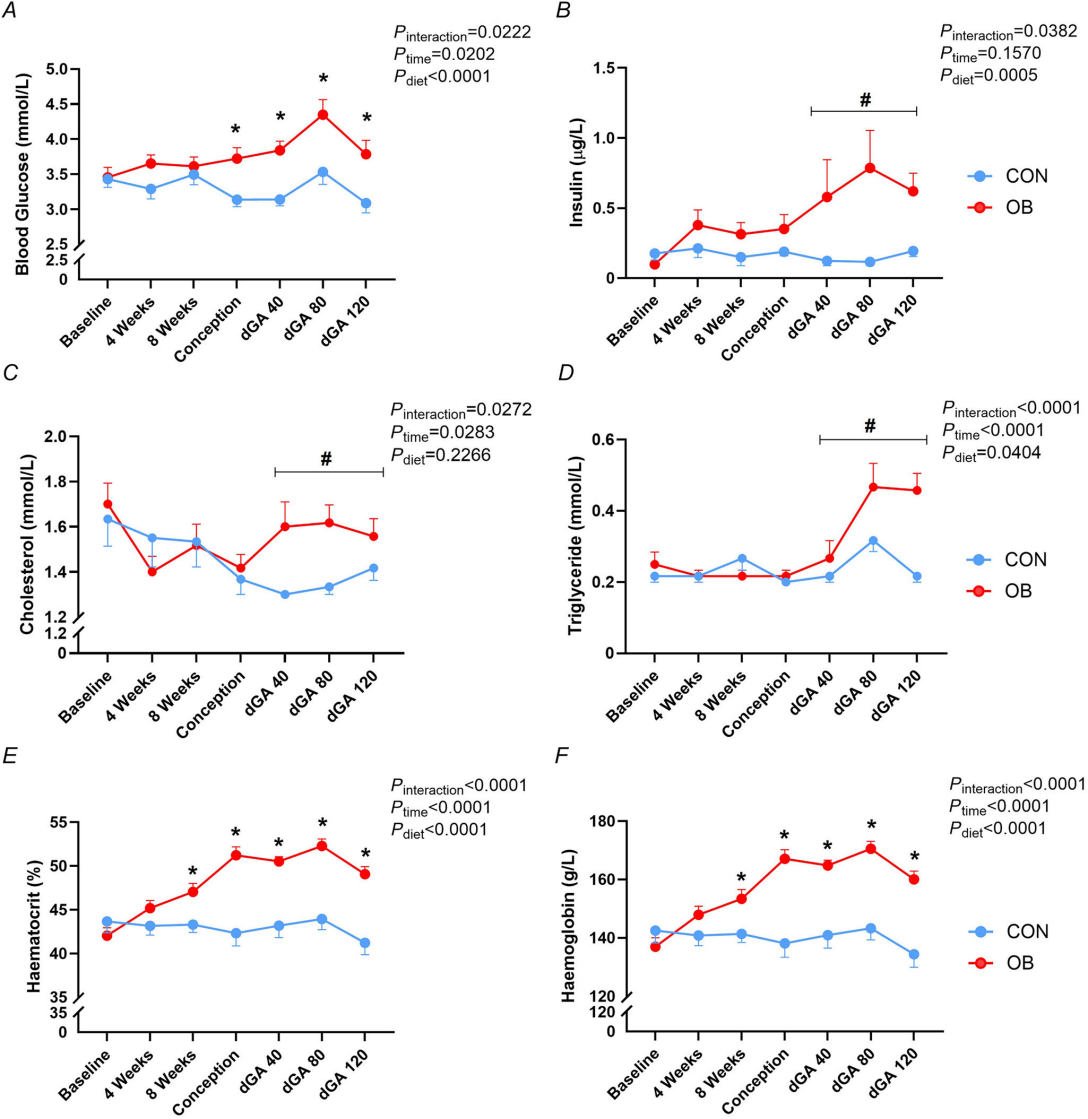
Maternal circulating metabolic markers *A*, ewe blood glucose; *B*, plasma insulin; *C*, plasma cholesterol; *D*, plasma triglycerides; *E*, haematocrit; and *F*, haemoglobin across the pre‐pregnancy, conception and pregnancy periods. All data are the mean ± SEM, with *n = *15–28 ewes per diet group, per time point for glucose, haemoglobin and haematocrit, and *n = *6–10 ewes per diet group, per time point for plasma markers. *P < 0.05; CON *vs*. OB comparison; Šídák's multiple comparisons test following Diet × Time interaction in two‐way repeated measures ANOVA. #*P* < 0.005; CON *vs*. OB AUC comparison during pregnancy; Šídák's multiple comparisons test following Diet × Pregnancy Status interaction in two‐way ANOVA.

Prior to pregnancy, there was no difference in blood glucose levels between ewe groups. However, OB ewes showed a significant Diet × Time interaction in blood glucose levels, where OB relative to CON mothers exhibited an 18–23% elevation in blood glucose, from conception through to 120 dGA (Fig. 2*A*). This was accompanied by a significant Diet × Time interaction in circulating insulin levels (Fig. 2*B*). Across the pre‐pregnancy period, the insulin area under the curve (AUC) was not significantly different between CON and OB ewes [0.38 ± 0.18 CON *vs*. 0.59 ± 0.27 OB; arbitrary units (AU), Fig. 2*B*]. However, OB relative to CON ewes showed a significantly greater insulin AUC throughout pregnancy (0.43 ± 0.11 AU CON *vs*. 1.85 ± 0.73 AU OB; *P* < 0.001). Similarly, total plasma cholesterol and triglyceride levels displayed Diet × Time interactions, where circulating lipid levels were no different between ewe groups in the pre‐pregnancy period. However, OB relative to CON ewes had significantly greater plasma lipid levels across pregnancy (Fig. 2*C* and *D*). Specifically, the AUC of both cholesterol (2.70 ± 0.09 AU CON *vs*. 3.20 ± 0.23 AU OB; *P* = 0.0013) and triglycerides (0.53 ± 0.06 AU CON *vs*. 0.83 ± 0.14 AU OB; *P* < 0.001) was significantly elevated in OB ewes during pregnancy (Fig. 2*C* and *D*). Further, OB relative to CON ewes showed significantly higher haematocrit and haemoglobin levels from 8 weeks of pre‐pregnancy feeding through to 120 dGA (Fig. 2*E* and *F*).

### Maternal *in vivo* systemic cardiovascular function and nutrient delivery

**Figure 3:**
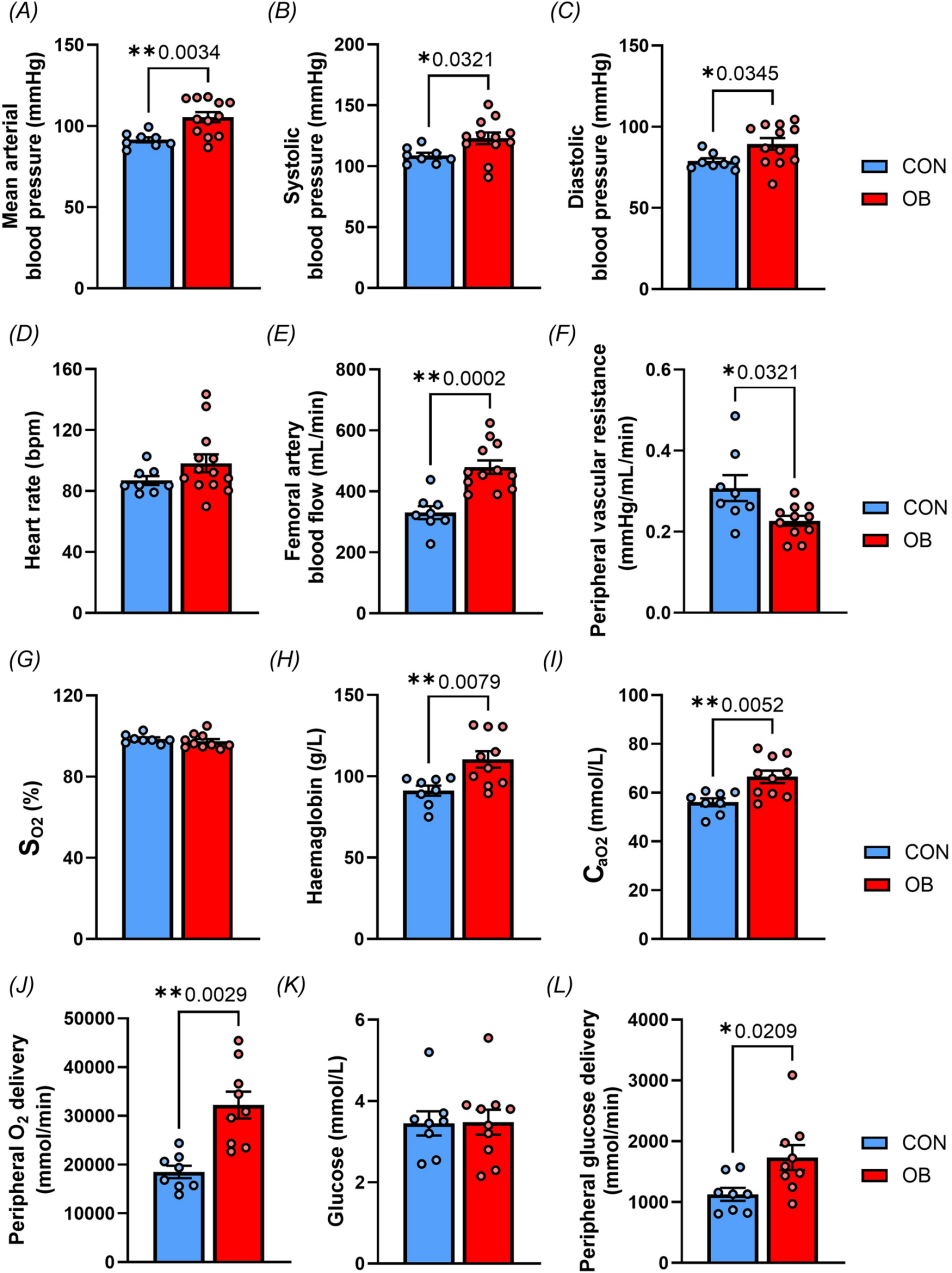
Maternal cardiovascular function in late pregnancy Maternal: *A*, mean blood pressure; *B*, systolic blood pressure; *C*, diastolic blood pressure; *D*, heart rate; *E*, femoral artery blood flow; *F*, femoral vascular resistance; *G*, oxygen saturation; *H*, haemoglobin; *I*, oxygen content; *J*, peripheral oxygen delivery; *K*, blood glucose; and *L*, peripheral glucose delivery in control (CON; blue) and obese (OB, red) ewes at 120–130 dGA. All data are the mean ± SEM, with *n = *7–12 ewes per diet group. *P < 0.05; **P < 0.01; ***P < 0.001; unpaired *t* test.

During basal measurements, OB relative to CON ewes were hypertensive, showing a 19% elevation in mean arterial blood pressure (Fig. 3*A*). This manifested as elevated systolic and diastolic pressure in OB ewes (Fig. 3*B* and *C*). While basal heart rate was similar between diet groups (Fig. 3*D*), maternal femoral arterial blood flow was elevated by 45% and femoral vascular resistance was reduced by 26% in OB relative to CON ewes (Fig. 3*E* and *F*). There was no difference in arterial oxygen saturation between ewe groups, but OB relative to CON ewes had significantly greater haemoglobin concentration (Fig. 3*G* and *H*). This resulted in arterial oxygen content and oxygen delivery in the femoral arterial circulation to be 18% and 74% greater in OB relative to CON ewes, respectively (Fig. 3*J* and *K*). In contrast, despite greater blood glucose levels in venous blood sampling from conception through to 120 dGA in non‐instrumented OB relative to CON ewes (Fig. 2*A*), arterial blood glucose levels in the instrumented ewes were no different between diet groups (Fig. 3*K*). However, glucose delivery to the femoral arterial circulation remained significantly elevated by 53% in OB relative to CON ewes (Fig. 3*L*).

### Maternal *in vivo* uterine vascular function and nutrient delivery

**Figure 4:**
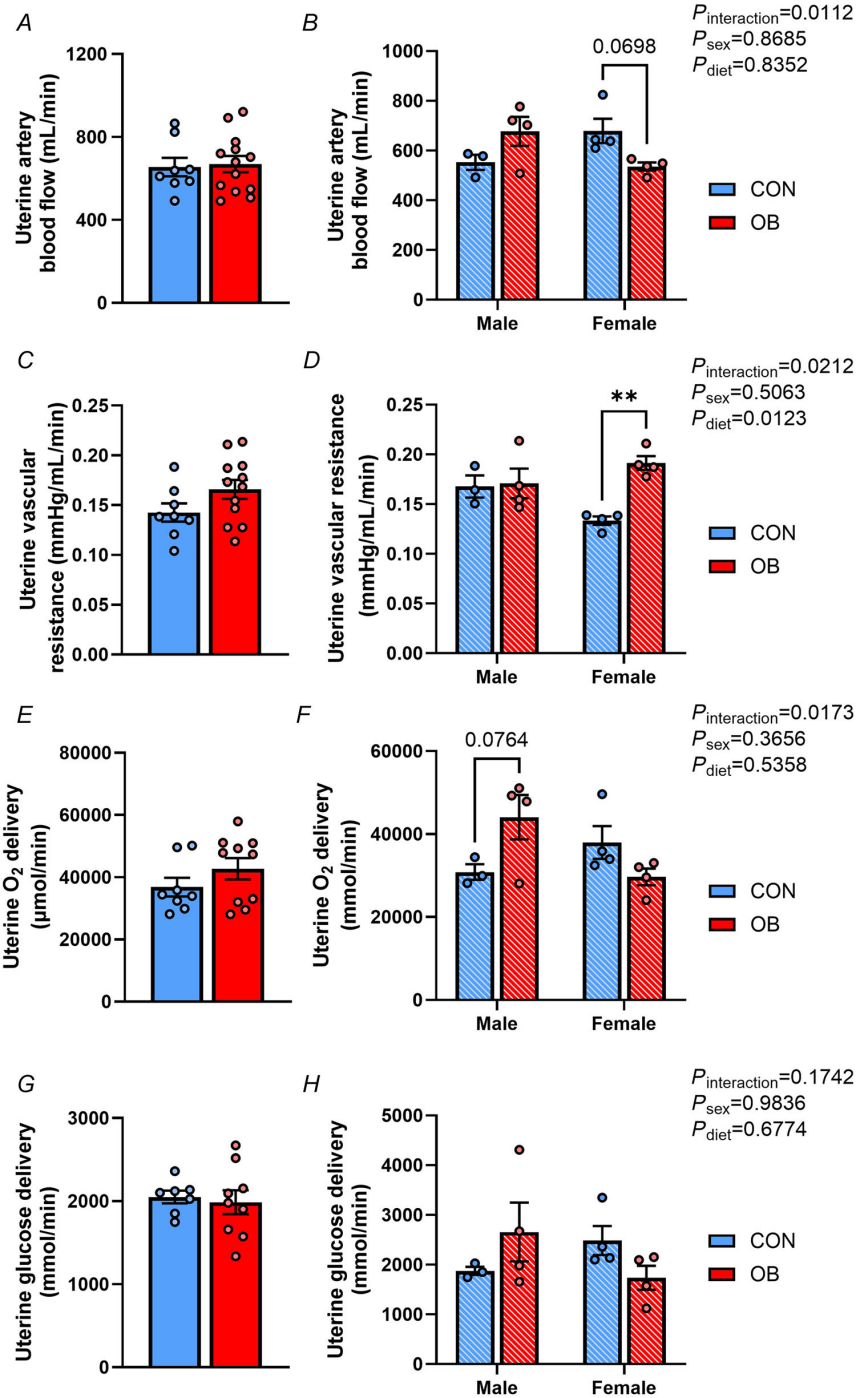
Uterine artery vascular function in late pregnancy Uterine artery: *A*, blood flow; *B*, blood flow separated by fetal sex; *C*, vascular resistance; *D*, vascular resistance separated by fetal sex; *E*, oxygen transport; *F*, oxygen transport separated by fetal sex; *G*, glucose transport; and *H*, glucose transport separated by fetal sex in control (*n = *8; blue) and obese (*n = *12; red) ewes at 120–130 dGA. All data are the mean ± SEM. **P < 0.005; CON *vs*. OB comparison; Šídák's multiple comparisons test following Diet × Fetal Sex interaction in two‐way ANOVA.

During basal measurements, OB relative to CON ewes had similar uterine artery blood flow, uterine vascular resistance, uterine oxygen delivery and uterine glucose delivery (Fig. 4*A*, *C*, *E* and *G*). However, in contrast to other outcome variables, there were significant Maternal Diet × Fetal Sex interactions in uterine blood flow and vascular resistance, whereby the uterine artery blood flow tended to be lower and uterine vascular resistance was 43% higher in OB relative to CON mothers carrying a female fetus (Fig. 4*B* and *D*). There was also a significant Maternal Diet × Fetal Sex interaction in uterine oxygen delivery, whereby OB mothers carrying a male fetus tended to have higher oxygen transport, relative to CON (Fig. 4*F*). Uterine glucose delivery was not impacted by maternal obesity or fetal sex (Fig. 4*H*).

### Maternal venous oxygenation, metabolic and electrolyte status at 130 dGA

Just prior to the animals being killed at 130 dGA, OB ewes showed an increase in venous $P_{O_{2}}$, but no difference in $P_{CO_{2}}$ or oxygen saturation compared to CON (Table 1). Corresponding to the longitudinal profile, OB relative to CON ewes had elevated haemoglobin and haematocrit (Table 1). OB mothers were also acidotic compared to CON, with a lower pH and a reduction in bicarbonate levels, resulting in a significant reduction in acid–base excess, relative to controls (Table 3). OB relative to CON ewes also had significantly greater values for circulating venous blood glucose, lactate, sodium and chloride levels (Table 1).

**Table 1:** **Maternal venous oxygenation, metabolic and electrolyte status at 130 dGA**.

Blood marker	CON		OB		*P*‐value
	Mean	SEM	Mean	SEM	
Oxygenation					
$P_{CO_{2}}$ (mmHg)	**41.7**	1.17	**41.7**	1.66	0.9870
$P_{O_{2}}$ (mmHg)	**41.1**	1.42	**46.4**	2.07	0.0429
$S_{O_{2}}$ (%)	**62.1**	2.10	**64.4**	3.34	0.5573
Haemoglobin (g/L)	**128**	2.47	**149**	3.67	<0.0001
Haematocrit (%)	**39.0**	0.78	**45.7**	1.13	<0.0001
**Acid–base status**					
pH	**7.36**	0.01	**7.29**	0.02	0.0063
Bicarbonate (mmol/L)	**22.4**	0.70	**18.8**	0.80	0.0019
Base Excess (mmol/L)	**−1.75**	0.91	**−6.64**	1.22	0.0031
**Metabolic status**					
Glucose (mmol/L)	**3.66**	0.21	**4.43**	0.23	0.0184
Lactate (mmol/L)	**7.10**	0.74	**9.87**	0.88	0.0219
**Electrolytes**					
K^+^ (mmol/L)	**5.33**	0.93	**4.66**	0.13	0.1041
Na^+^ (mmol/L)	**149**	0.60	**151**	0.45	0.0116
Ca^2+^ (mmol/L)	**1.15**	0.02	**1.14**	0.02	0.8857
Cl^−^ (mmol/L)	**108**	0.51	**110**	0.49	0.0098

*Note*: All data are the mean ± SEM. *P* values are derived from unpaired *t* tests between control (CON; *n = *18) and obese (OB; *n = *17 −18) ewes at 130 dGA.

### Maternal, fetal and placental biometry at post‐mortem

At post‐mortem, at 130 dGA, OB relative to CON ewes showed greater adiposity (Table 2 and Fig. 1*D* and *E*), and increased mass in several metabolic and endocrine organs, including an increase in heart, adrenal gland, kidney, liver, spleen and thyroid gland weight (Table 2). When organ weights were expressed per kg of maternal body weight, relative heart rate was significantly lower, and relative pericardial and perirenal fat deposits remained significantly elevated in OB relative to CON ewes, consistent with increased fat mass preferentially contributing to the increase in body weight (Table 2).

**Table 2:** Maternal biometry at 130 dGA

Maternal outcome	CON		OB		*P*‐value
	Mean	SEM	Mean	SEM	
dGA at post‐mortem	**130**	0.52	**129**	0.37	0.2863
Body weight (kg)	**49.0**	1.01	**60.2**	1.25	<0.0001
**Organ weight (g)**					
Heart	**225**	7.55	**245**	4.22	0.0233
Pericardial fat	**82.8**	7.22	**178.0**	11.99	<0.0001
Adrenal	**2.93**	0.23	**3.62**	0.38	0.1123
Kidney	**52.8**	2.39	**61.6**	3.61	0.0437
Perirenal fat	**163**	26.6	**459**	48.7	<0.0001
Pancreas	**38.8**	3.31	**44.2**	4.51	0.3328
Liver	**640**	20.8	**768**	40.5	0.0075
Spleen	**269**	22.3	**425**	38.2	0.0009
Thyroid	**1.87**	0.12	**2.38**	0.22	0.0393

Relative organ weight (g/kg BW)					
Heart	**4.62**	0.16	**4.11**	0.15	0.022
Pericardial fat	**1.66**	0.16	**2.93**	*0.17*	<0.0001
Adrenal	**0.06**	0.00	**0.06**	0.01	0.8678
Kidney	**1.09**	0.05	**1.02**	0.05	0.2608
Perirenal fat	**3.23**	0.47	**7.49**	0.67	<0.0001
Pancreas	**0.79**	0.06	**0.75**	0.08	0.6123
Liver	**13.1**	0.31	**12.8**	0.68	0.5893
Spleen	**5.59**	0.45	**7.11**	0.67	0.0566
Thyroid	**0.04**	0.00	**0.04**	0.00	0.9883

*Note*: All data are the mean ± SEM. *P* values are derived from unpaired *t* tests between control (CON; *n = *12–26) and obese (OB; *n = *15–28) ewes at 130 dGA. dGA, days gestational age; BW, body weight.

**Figure 5:**
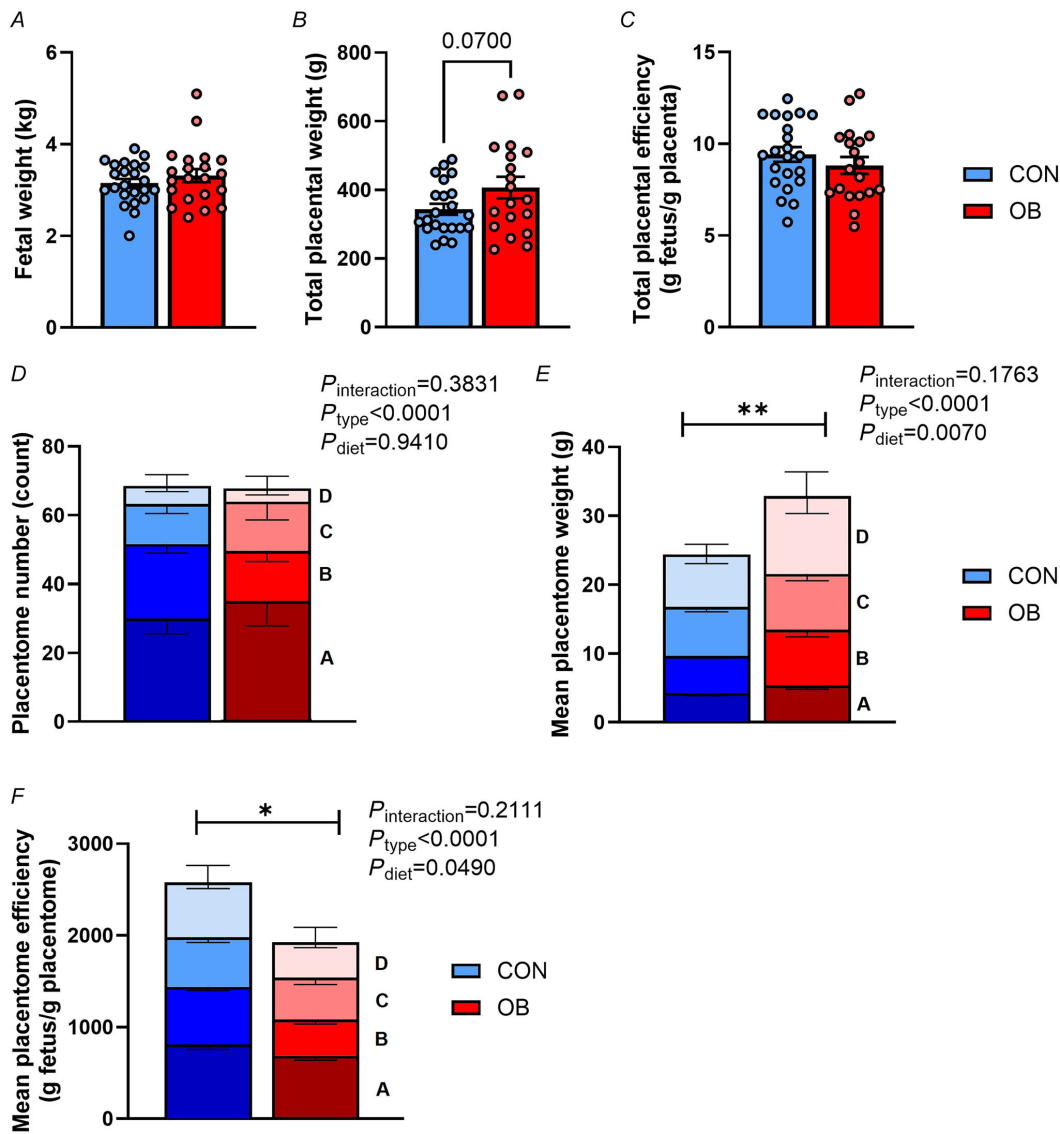
Fetal and placental phenotype at 130 dGA *A*, fetal weight; *B*, total placental weight; *C*, total placental efficiency; *D*, placentome number; *E*, mean placentome weight; and *F*, mean placentome efficiency in control (CON; blue; *n = *21) and obese (OB; red; *n = *14) pregnancies at 130 dGA. All data are the mean ± SEM. No fetal sex differences were observed (*P* > 0.05; two‐way ANOVA), so male and female values are combined. *P < 0.05, **P = 0.01; Diet effect in mixed effects model.

At post‐mortem, at 130 dGA, fetal body weight, placental weight and placental efficiency (fetal body weight/placental weight) were similar in CON and OB groups (Fig. 5*A*–*C*). When placentomes were typed, the mean overall placentome type weight but not number was significantly elevated in OB relative to CON ewes (Fig. 5*D* and *E*). Therefore, placentomal efficiency was significantly reduced in OB relative to CON ewes (Fig. 5*F*). At post‐mortem, fetuses from OB relative to CON ewes also showed an increase in CRL, BPD and femur length, resulting in significantly lower values for PI (Table 3). Values for absolute but not relative heart, lung, kidney and perirenal fat were also significantly increased in fetuses from OB relative to CON ewes (Table 3).

**Table 3:** Fetal biometry at 130 dGA

Fetal outcome	CON		OB		*P*‐value
	Mean	SEM	Mean	SEM	
dGA at post‐mortem	**130**	0.52	**129**	0.37	0.2863
Body weight (kg)	**3.15**	0.09	**3.32**	0.14	0.3292
**Morphometry**					
Crown–rump length (cm)	**42.4**	0.56	**44.2**	0.64	0.0329
Biparietal diameter (cm)	**9.56**	0.26	**11.05**	0.35	0.0013
Abdominal circumference (cm)	**29.5**	0.61	**29.8**	1.37	0.8581
Femur length (cm)	**11.3**	0.26	**12.2**	0.29	0.0390
BMI [weight (kg)/CRL (cm)^2^]	**17.6**	0.42	**16.8**	0.40	0.2183
PI [weight (g) × 100)/CRL (cm)^3^]	**4.16**	0.12	**3.81**	0.10	0.0337
**Organ weight (g)**					
Heart	**22.3**	0.71	**24.5**	0.94	0.0604
Brain	**41.8**	1.03	**44.8**	1.10	0.0522
Right lung	**41.7**	1.41	**47.9**	2.67	0.0379
Left lung	**28.2**	1.01	**32.3**	2.09	0.0665
Pericardial fat	**7.54**	0.44	**8.50**	0.63	0.2117
Adrenal	**0.17**	0.01	**0.20**	0.01	0.4407
Kidney	**8.51**	0.32	**10.38**	0.82	0.0212
Perirenal fat	**5.90**	0.28	**7.06**	0.47	0.0328
Pancreas	**2.36**	0.15	**2.72**	0.21	0.1675
Liver	**82.0**	3.61	**95.7**	7.16	0.0884
Spleen	**5.07**	0.21	**5.86**	0.41	0.0705
Thyroid	**0.33**	0.02	**0.36**	0.03	0.5150
**Relative organ weight (g/kg BW)**					
Heart	**7.11**	0.17	**7.51**	0.29	0.2243
Brain	**13.6**	0.51	**13.6**	0.41	0.9149
Right lung	**12.3**	0.72	**13.8**	0.42	0.1242
Left lung	**8.34**	0.49	**9.28**	0.31	0.1467
Pericardial fat	**2.39**	0.12	**2.53**	0.12	0.4238
Adrenal	**0.06**	0.00	**0.06**	0.00	0.6254
Kidney	**2.55**	0.18	**2.93**	0.14	0.1634
Perirenal fat	**1.84**	0.07	**1.99**	0.09	0.2158
Pancreas	**0.74**	0.04	**0.73**	0.07	0.8922
Liver	**26.6**	1.45	**28.6**	1.92	0.3124
Spleen	**1.65**	0.08	**1.70**	0.09	0.6808
Thyroid	**0.10**	0.01	**0.10**	0.01	0.5753

*Note*: All data are the mean ± SEM. *P* values are derived from unpaired *t* tests between control (CON; *n = *19–23) and obese (OB; *n = *12–21) fetuses at 130 dGA. There was no significant effect of fetal sex on any outcome (*P* > 0.05; two‐way ANOVA), so male and female data are combined. BMI, body mass index; PI, ponderal index.

### Uterine artery vascular reactivity measured *ex vivo* via *in vitro* wire myography

**Figure 6:**
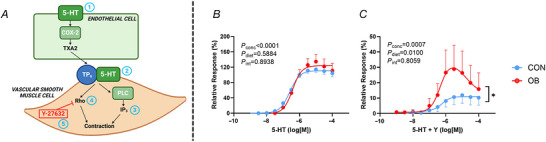
Uterine artery vascular constrictor reactivity at dGA 130 *A*, diagram of vasoconstrictor pathways in the uterine artery: serotonin (5‐HT) can induce vasoconstriction via endothelium‐dependent thromboxane A2 (TXA2) production in endothelial cells (1) or by direct action on vascular smooth muscle cells (2). Activation of phospholipase C (PLC) produces inositol triphosphate (IP3) (3), which releases Ca^2+^ from the sarcoplasmic reticulum to activate myosin light chain kinase, thereby stimulating vascular smooth muscle cell contraction. Alternatively, Rho kinase can directly phosphorylate myosin light chain and inhibit myosin light chain phosphatase activity to stimulate contraction (4). Rho kinase activity is blocked by the synthetic inhibitor, Y27632 (Y) (5). Uterine artery concentration response curves to: *B*, serotonin (5‐HT); and *C*, 5‐HT+Y in control (CON; blue) and obese (OB; red) ewes at 130 dGA. All data are the mean ± SEM with *n = *10–13 ewes per diet group. *P < 0.05 Diet effect in two‐way ANOVA.

Vascular smooth muscle constriction induced by 5‐HT involves Rho kinase (Fig. 6*A*). Uterine vascular constrictor reactivity to 5‐HT was similar before, but significantly attenuated following blockade with the synthetic Rho kinase inhibitor Y27632 in CON relative to OB ewes, independent of the sex of the fetus (Fig. 6*B* and C). This suggests that OB ewes rely more on a Rho kinase‐independent mechanism to mediate the constrictor response to 5‐HT in the uterine vasculature.

**Figure 7:**
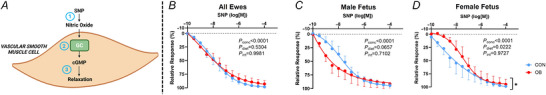
Uterine artery vascular smooth muscle‐dependent dilator reactivity at  130 gDA *A*, diagram of vascular smooth muscle‐dependent vasodilator pathways in the uterine artery: sodium nitroprusside (SNP) breaks down to produce nitric oxide (1). This stimulates guanylate cyclase (GC) in vascular smooth muscle cells to activate cyclic GMP (cGMP) (2). cGMP reduces intracellular calcium levels, which induces vasorelaxation (3). Uterine artery concentration response curves to sodium SNP in: *B*, all ewes; *C*, ewes carrying a male fetus; and *D*, ewes carrying a female fetus. All data are the mean ± SEM with *n = *10–13 ewes per diet group in combined ewe analysis, *n = *4–7 ewes per group in separate fetal sex analysis. *P < 0.05 Diet effect in two‐way ANOVA.

Uterine vascular dilator reactivity to the smooth muscle‐dependent agonist SNP (Fig. 7*A*) was similar in OB relative to CON ewes when all ewes were included (Fig. 7*B*). However, there was a significant Maternal Diet × Fetal Sex interaction (*P* = 0.0039; Fig. 7*B*), whereby ewes carrying a female, but not male, fetus displayed impaired uterine artery relaxation in response to lower doses of SNP relative to CON (Fig. 7*C* and *D*).

**Figure 8:**
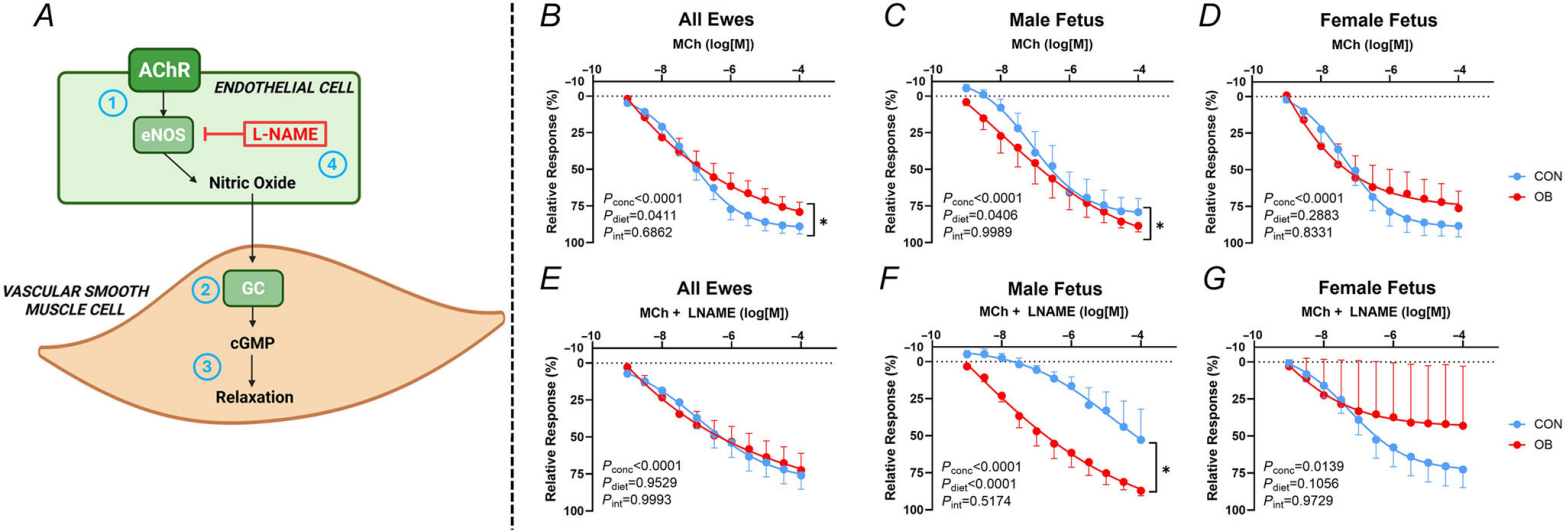
Uterine artery vascular endothelial‐dependent dilator reactivity at  130 gDA *A*, diagram of vascular endothelial‐dependent vasodilator pathways in the uterine artery: methacholine (MCh) binds to vascular endothelial cell acetylcholine receptors (AChR) to induce endothelial nitric oxide synthase (eNOS) (1). This produces nitric oxide, which stimulates guanylate cyclase (GC) in vascular smooth muscle cells to activate cyclic GMP (cGMP) (2). cGMP reduces intracellular calcium levels, which induces vasorelaxation (3). Administration of l‐NAME directly blocks eNOS action in endothelial cells, thereby preventing MCh‐induced vasodilatation via this pathway (4). Uterine artery concentration response curves to: *B*, MCh in all ewes; *C*, MCh in ewes carrying a male fetus; *D*, MCh in ewes carrying a female fetus; *E*, MCh + l‐NAME in all ewes; *F*, MCh + l‐NAME in ewes carrying a male fetus; and *G*, MCh + l‐NAME in ewes carrying a female fetus, in control (CON; blue) and obese (OB; red) ewes at 130 dGA. All data are the mean ± SEM with *n = *10–13 ewes per diet group in combined ewe analysis, *n* = 2–7 ewes per group in separate fetal sex analysis. *P < 0.05 Diet effect in two‐way ANOVA.

Uterine artery vascular dilator reactivity to the endothelium‐dependent agonist methacholine (MCh; Fig. 8*A*) was significantly impaired when all ewes were included before but not after blockade with l‐NAME in OB relative to CON ewes (Fig. 8*B* and *E*). However, there was a significant interaction between maternal diet and fetal sex. OB ewes carrying a male fetus had greater vasorelaxation to MCh before (Fig. 8*C*) and following l‐NAME (Fig. 8*F*), while OB ewes carrying a female fetus were unaffected (Fig. 8*D* and *G*). This suggests that OB ewes carrying a male fetus have greater NO‐independent mechanisms mediating the uterine vasodilator response to MCh.

## Discussion

This study introduces a novel ovine model of diet‐induced obesity during pregnancy, where ewes with obesity display increased adiposity, hyperglycaemia, hyperinsulinaemia, hyperlipidaemia and hypertension, constituting cardiometabolic dysfunction typical of the metabolic syndrome. Relative to lean controls, ewes with obesity also showed increased haemoglobin throughout the study period, with elevated femoral blood flow and enhanced peripheral oxygen transport near term. Maternal uterine artery function was significantly affected by obesity, showing increased vascular resistance *in vivo*. This was explained in part by a greater reactivity to Rho kinase‐independent constrictor mechanisms stimulated by 5‐HT and reduced dilator responses to the endothelium‐dependent agonist MCh *ex vivo*. Effects on maternal uterine vasoreactivity in obese ewes varied according to the sex of the fetus, showing reduced smooth‐muscle‐dependent vasorelaxation to SNP *ex vivo* in mothers with obesity carrying a female, but not male, fetus. Conversely, mothers with obesity carrying a male, but not female, fetus showed greater NO‐independent mechanisms mediating the uterine vasodilator response to MCh *ex vivo*. These cardiometabolic disturbances occurred with evidence of decreased placental efficiency and alterations in fetal growth, promoting a thin‐for‐length phenotype with a reduced PI in fetuses of both sexes in ewes with obesity.

Ewes in the obese group gained weight steadily throughout the pre‐pregnancy feeding period and entered pregnancy substantially heavier than controls. Moreover, expected metabolic disturbances associated with excess adiposity, including hyperglycaemia, hyperinsulinaemia and hyperlipidaemia, developed in the obese ewes with pregnancy onset and progression. This reinforces the concept that pregnancy itself is a metabolic challenge that exacerbates underlying subclinical pathologies, as noted in cases of gestational diabetes mellitus (Catalano, 2014).

Interestingly, pregnant ewes with obesity also exhibited a striking increase in haemoglobin and haematocrit relative to controls, which started after 8 weeks of obesogenic feeding and persisted throughout pregnancy. Elevated haemoglobin has been noted in recent cohorts of pregnant women with obesity relative to lean‐weight women (Elmugabil et al., 2017; Eltayeb et al., 2023) and higher haemoglobin levels, particularly in the first trimester, have been positively associated with elevated BMI and gestational diabetes mellitus onset in larger‐scale population studies (Sissala et al., 2022; Wang et al., 2018). The mechanisms underlying this elevated haemoglobin profile in women with obesity are unclear, but may be driven by maternal insulin status, since insulin can stimulate erythropoietin production by activating the HIF‐1 signalling cascade (Treins et al., 2002) and/or by acting directly as a growth factor (Masuda et al., 1997).

The elevated maternal haemoglobin levels in obese ewes may also contribute to their hypertensive phenotype, since heightened haemoglobin levels in the first trimester have been positively associated with the onset of pregnancy‐induced hypertension (Abumohsen et al., 2021; Aghamohammadi et al., 2011) and pre‐eclampsia (Wang et al., 2018) in otherwise healthy women. Mechanisms linking elevated haemoglobin to hypertension may include an increase in blood viscosity, promoting enhanced peripheral vascular resistance according to Poiseuille's Law and, thereby, an increase in cardiac afterload (Çınar et al., 1999; Letcher et al., 1981). Since diastolic arterial blood pressure reflects downstream peripheral vascular resistance, its elevation in pregnant ewes with obesity supports this contention. Additionally, ewes with obesity in our study had greater extracellular fluid (ECF) concentrations of sodium and reduced heart weight in relation to their total body weight compared to lean ewes. Greater sodium will lead to ECF volume expansion, increasing total fluid volume, which may have contributed to the raised blood pressure in pregnant ewes with obesity. A relatively smaller heart to perfuse a larger body, combined with higher blood viscosity and cardiac afterload, will contribute to higher cardiac workload in pregnant ewes with obesity. Significantly greater systolic pressure in pregnant ewes with obesity supports this contention. Increased cardiac workload, hypertension and higher haemoglobin levels are all strongly associated with long‐term cardiometabolic disease risk (Honigberg et al., 2019; Mehta & Dubrey, 2009; Tapio et al., 2021), and so may perpetuate cardiometabolic dysfunction in mothers with obesity long after birth.

Peripheral vascular function was altered substantially in pregnant ewes with obesity near term, with elevated femoral blood flow, reduced femoral vascular resistance, and enhanced oxygen and glucose supply to peripheral vascular beds, relative to controls. Elevated blood flow in the peripheral vasculature has also been previously noted in pregnant women with obesity (Dutta et al., 2020) and may be facilitated by the high‐volume to low‐resistance circulation profile noted in obese relative to control pregnancies (Vonck et al., 2019). However, sustained elevations in blood flow can induce endothelial dysfunction by the activation of shear stress pathways, leading to vascular remodelling and changes to vascular tone (Lu & Kassab, 2011).

The elevated oxygen delivery in pregnant ewes with obesity is somewhat counter‐intuitive, since obesity is typically associated with adipose tissue hypoxia in non‐pregnant individuals (Mirabelli et al., 2024; Ye, 2009), and placental hypoxia in rodent models of obese pregnancy (Fernandez‐Twinn et al., 2017; Wallace et al., 2019). While the elevated haemoglobin content in ewes with obesity clearly underlies the elevated oxygen transport findings, the haemoglobin‐driven hyperviscosity may also reduce perfusion at the microvascular level (Mirabelli et al., 2024). Therefore, there may be a disconnect between oxygen transport levels based on arterial conduit calculations, and those occurring at terminal arterial branches. Moreover, the placenta uses 40–70% of uterine oxygen supply to sustain its own high metabolic demands, in both humans and sheep (Carter, 2000; Sferruzzi‐Perri et al., 2019). Placental oxygen consumption is also positively associated with oxygen supply *in vitro* (Schneider, 2000). The elevated uterine artery oxygen transport in obese ewes could therefore be associated with increased placental oxygen consumption, which typically reduces placental efficiency. This may explain why we found an increase in placentomal weight, but no corresponding increase to fetal weight in the obese group, although assessment of tissue‐specific oxygenation levels and placental oxygen metabolism pathways would be required to confirm this.

Uterine artery function was altered by maternal obesity in a fetal sex‐specific manner, with elevated uterine artery vascular resistance and impaired smooth muscle‐dependent relaxation capacity in pregnant ewes with obesity carrying a female fetus. In healthy pregnancy, the uterine vascular bed is subject to critical remodelling, resulting in lower uterine vascular resistance towards term, which facilitates maximal oxygen and nutrient supply to the growing fetus (Osol & Mandala, 2009). Therefore, ewes with obesity carrying a female fetus appear more vulnerable to reduced uterine nutritional supply, and our *ex vivo* data suggest that this occurs through vascular smooth muscle alterations. Interestingly, obese ewes carrying a male fetus did not exhibit any significant *in vivo* uterine artery changes relative to controls. Rather, our *ex vivo* data suggest that mothers with obesity carrying a male fetus showed greater NO‐independent mechanisms mediating the uterine vasodilator response to MCh *ex vivo*. This may act as a compensatory mechanism to maintain appropriate uterine artery vasomotor function, which is consistent with other evidence of maternal obesity leading to recruitment of NO‐independent compensatory mechanisms for vasodilatation in a mouse model of pre‐eclampsia (Binder et al., 2025). Interestingly, the elevated haemoglobin levels in OB mothers may contribute to this adaptation by reducing NO bioavailability, since haemoglobin can oxidize NO to generate nitrates and methaemoglobin (Gow et al., 1999; Keszler et al., 2008). We have not assessed nitrate levels in the present study, but further studies into NO metabolism in this model appear warranted considering these uterine artery adaptations.

Previous studies have found that women carrying a male fetus exhibit a higher uterine artery Doppler pulsatility index compared to those carrying a female fetus (Broere‐Brown et al., 2016; Teulings et al., 2020). Teulings et al. (2020) further demonstrated that maternal obesity increases uterine pulsatility index, but this occurred independently of fetal sex effects (Teulings et al., 2020). To our knowledge, our study is the first to assess the interaction between maternal obesity and fetal sex on uterine artery function. Our model found significant Maternal Diet × Fetal Sex interactions in most uterine artery outcomes. Therefore, further investigation into the importance of maternal–fetal crosstalk in obese pregnancy is merited, and our study highlights specific mechanistic pathways to target. While there was no difference in fetal weight between diet groups at 130 dGA, fetuses exposed to maternal obesity showed a reduced PI. Fetal growth outcomes in obese pregnancy are complex and somewhat heterogenic between diverse populations and models, and different studies have reported fetal overgrowth, undergrowth or no change to fetal weight with maternal obesity (Crew et al., 2016; Langley‐Evans et al., 2022; Lewandowska, 2021). Our model reproducing decreased placental efficiency with fetuses that are thin‐for‐their‐length is interesting, as it is this phenotype in offspring of complicated pregnancy that has been linked to future cardiovascular risk (Eriksson et al., 2001).

This study provides a novel and translatable model for maternal obesity, but also has several limitations. While the work is generally well powered, several of the sex‐specific comparisons in the myography experiments are limited by low *n* values. This is due to the experimental time and complexity associated with ovine models, which must be balanced with their translational advantages. We were also unable to account for the specific number of previous pregnancies in our ewes, although this will be between one and two, given their age and the tightly controlled seasonality of sheep breeding. Parity is emerging as an important factor in cardiovascular disease development in women (Klingberg et al., 2017; Li et al., 2019), so future studies should consider the interaction between parity and gestational complications, including maternal obesity.

In summary, this study presents a robust model of maternal obesity in pregnancy that exhibits cardiometabolic dysfunction near term and recapitulates key aspects of the human phenotype, including increased adiposity, hyperglycaemia, hyperinsulinaemia, hyperlipidaemia and hypertension in the mother. Further, we provide mechanistic insights into fetal sex‐specific uterine vascular bed dysfunction that may have significant implications for the maternal cardiovascular adaptation to pregnancy, and the long‐term maternal and offspring cardiovascular health after pregnancy complicated by obesity. The fetal sex‐specific changes to maternal uterine artery function in obese ewes also highlight the importance of fetal‐to‐maternal communication in regulating maternal vascular function during pregnancy.

## Additional information

## Competing interests

No competing interests are declared.

## Author contributions

Study concept and funding acquisition: D.A.G., S.E.O., M.P.M. Model optimization and study design: D.A.G., R.C.C., S.G.F. *In vivo* experiments and tissue generation: R.C.C., A.L.K.C., Y.N., S.G.F., C.L.R.C., S.H.D., D.A.G. Lab work and biochemical assays: R.C.C., A.L.K.C. Data analyses and results interpretation: R.C.C., A.L.K.C., D.A.G. Manuscript draft: R.C.C., D.A.G. Manuscript revision and approval: R.C.C., A.L.K.C., Y.N., S.G.F., C.L.R.C., S.H.D., M.P.M., S.E.O., D.A.G.

## Funding

This work was supported by the UK MRC [MR/V03362X/1]. R.C.C. was supported by the Cambridge BHF Centre of Research Excellence and the Isaac Newton Trust.

## Supporting information


Peer Review History

## Data Availability

Data will be made available by the authors upon reasonable request.
