# Supplementary material for: Maternal cardiometabolic dysfunction and fetal sex‐specific alterations to uterine vascular reactivity in an ovine model of obesity during pregnancy
Source: J Physiol. 2026 May 22;604(12):5011–30. doi: 10.1113/JP290766 (PMC13267682; doi:10.1113/JP290766)
Supplement: Supplementary file 1 — Peer Review History [file TJP-604-5011-s001.pdf]

The Journal of Physiology

Maternal cardiometabolic dysfunction and fetal sex-specific alterations to uterine vascular reactivity in an ovine model of obesity during pregnancy

Rachael C Crew, Anna LK Cochrane, Youguo Niu, Sage G Ford, Clement L R Cahen, Skaai H Davison, Michael P. Murphy, Susan E Ozanne, and Dino A Giussani
DOI: 10.1113/JP290766

Corresponding author(s): Rachael Crew (rc873@cam.ac.uk)

The following individual(s) involved in review of this submission have agreed to reveal their identity: Clare Reynolds (Referee #1); Deborah M Sloboda (Referee #2)

Review Timeline:

Submission Date:	08-Jan-2026
Editorial Decision:	11-Feb-2026
Revision Received:	27-Feb-2026
Editorial Decision:	17-Mar-2026
Revision Received:	17-Apr-2026
Accepted:	22-Apr-2026

Senior Editor: Laura Bennet

Reviewing Editor: Christopher Lear

Transaction Report:

(Note: With the exception of the correction of typographical or spelling errors that could be a source of ambiguity, letters and reports are not edited. Depending on transfer agreements, referee reports obtained elsewhere may or may not be included in this compilation. Referee reports are anonymous unless the Referee chooses to sign their reports.)

1st Editorial Decision 11-Feb-2026

Dear Dr Giussani,

Re: JP-RP-2026-290766 "**Maternal cardiometabolic dysfunction and fetal sex-specific alterations to uterine vascular reactivity in an ovine model of obesity during pregnancy**" by Rachael C Crew, Anna LK Cochrane, Youguo Niu, Sage G Ford, Clement L R Cahen, Skaai H Davison, Michael P. Murphy, Susan E Ozanne, and Dino A Giussani

Thank you for submitting your manuscript to The Journal of Physiology. It has been assessed by a Reviewing Editor and by 2 expert referees and we are pleased to tell you that it is acceptable for publication following satisfactory revision.

The referee reports are copied at the end of this email.

Please address all the points raised and incorporate all requested revisions or explain in your Response to Referees why a change has not been made. We hope you will find the comments helpful and that you will be able to return your revised manuscript within 4 weeks. If you require longer than this, please contact journal staff: jp@physoc.org.

Your revised manuscript should be submitted online using the link in your Author Tasks Link Not Available. This link is accessible via your account as Corresponding Author; it is not available to your co-authors. If this presents a problem, please contact journal staff (jp@physoc.org). Image files from the previous version are retained on the system. Please ensure you replace or remove any files that are being revised.

If you do not wish to submit a revised version of your manuscript, you must inform our journal staff (jp@physoc.org) or reply to this email to request withdrawal. Please note that a manuscript must be formally withdrawn from the peer review process at one journal before it may be submitted to another journal.

TRANSPARENT PEER REVIEW POLICY: To improve the transparency of its peer review process The Journal of Physiology publishes online, as supporting information, the peer review history of all articles accepted for publication. Readers will have access to decision letters, including Editors' comments and referee reports, for each version of the manuscript, as well as any author responses to peer review comments. Referees can decide whether or not they wish to be named on the peer review history document.

ABSTRACT FIGURES: Authors are expected to use The Journal's premium BioRender account to create/redraw their Abstract Figures. Information on how to access this account is here: https://physoc.onlinelibrary.wiley.com/journal/14697793/biorender-access.

This will enable Authors to create and download high-resolution figures. If authors have used the free BioRender service, they can use the instructions provided in the link above to download a high-resolution version suitable for publication.

The link provided should only be used for the purposes of this submission. Authors will be charged for figures created on this account if they are not related to this manuscript submission.

LANGUAGE EDITING AND SUPPORT FOR PUBLICATION: If you would like help with English language editing, or other article preparation support, Wiley Editing Services offers expert help, including English Language Editing, as well as translation, manuscript formatting, and figure formatting at www.wileyauthors.com/eeo/preparation. You can also find resources for Preparing Your Article for general guidance about writing and preparing your manuscript at www.wileyauthors.com/eeo/prepresources.

RIGOUR AND REPRODUCIBILITY: We recommend authors consult The Journal's Rigour and Reproducibility requirements to ensure that their manuscript is suitable for publication: https://physoc.onlinelibrary.wiley.com/pb-assets/hub-assets/physoc/documents/TJP-Rigour-and-Reproducibility-Requirements.pdf

REVISION CHECKLIST:

Check that your Methods section conforms to journal policy: https://jp.msubmit.net/cgi-bin/main.plex?form_type=display_requirements#methods

Check that data presented conforms to the statistics policy: https://jp.msubmit.net/cgi-bin/main.plex?form_type=display_requirements#statistics

Upload a full Response to Referees file. To create your 'Response to Referees' copy all the reports, including any comments from the Senior and Reviewing Editors, into a Microsoft Word, or similar, file and respond to each point, using font or background colour to distinguish comments and responses and upload as the required file type.

Please upload two versions of your manuscript text: one with all relevant changes highlighted and one clean version with no changes tracked. The manuscript file should include all tables and figure legends, but each figure/graph should be uploaded as separate, high-resolution files. The journal is now integrated with Wiley's Image Checking service. For further details, see: https://www.wiley.com/en-us/network/publishing/research-publishing/trending-stories/upholding-image-integrity-wileys-

image-screening-service

You may also upload:

- 'Potential Cover Art' for consideration as the issue's cover image
- Appropriate Supporting Information (Video, audio or data set: see https://jp.msubmit.net/cgi-bin/main.plex?form_type=display_requirements#supp).

We look forward to receiving your revised submission.

If you have any queries, please reply to this email and we will be pleased to advise.

Yours sincerely,

Laura Bennet
Senior Editor
The Journal of Physiology

REQUIRED ITEMS FOR REVISION

- Author photo and profile. First or joint first authors are asked to provide a short biography (no more than 100 words for one author or 150 words in total for joint first authors) and a portrait photograph. These should be uploaded and clearly labelled together in a Word document with the revised version of the manuscript. See Information for Authors for further details.

- You must start the Methods section with a paragraph headed Ethical approval (https://jp.msubmit.net/cgi-bin/main.plex?form_type=display_requirements#methods).

Research must comply with The Journal's policies regarding animal experiments (https://physoc.onlinelibrary.wiley.com/hub/animal-experiments) and adherence to these policies must be stated in the manuscript.

Authors should confirm in their Methods section that their experiments were carried out according to the guidelines laid down by their institution's animal welfare committee, including an ethics approval reference number. The Methods section must contain a statement about access to food, water and housing, details of the anaesthetic regime: anaesthetic used, dose and route of administration, and method of killing the experimental animals.

- Your manuscript must include a complete Additional Information section, including competing interests; funding; author contributions and acknowledgements.

- Please upload separate high-quality figure files via the submission form.

- Papers must comply with the Statistics Policy: https://jp.msubmit.net/cgi-bin/main.plex?form_type=display_requirements#statistics.

In summary:

- If n {less than or equal to} 30, all data points must be plotted in the figure in a way that reveals their range and distribution. A bar graph with data points overlaid, a box and whisker plot or a violin plot (preferably with data points included) are acceptable formats.

- If n > 30, then the entire raw dataset must be made available either as supporting information, or hosted on a not-for-profit repository, e.g. FigShare, with access details provided in the manuscript.

- 'n' clearly defined (e.g. x cells from y slices in z animals) in the Methods. Authors should be mindful of pseudoreplication.

- All relevant 'n' values must be clearly stated in the main text, figures and tables.

- The most appropriate summary statistic (e.g. mean or median and standard deviation) must be used. Standard Error of the Mean (SEM) alone is not permitted.

- Exact p values must be stated. Authors must not use 'greater than' or 'less than'. Exact p values must be stated to three significant figures even when 'no statistical significance' is claimed.

- Please include an Abstract Figure file and an Abstract Figure legend. An appropriate figure legend, which should not exceed 150 words in length, should be included in the main manuscript file. The Abstract Figure is a piece of artwork designed to give readers an immediate understanding of the research and should summarise the main conclusions. If possible, the image should be easily 'readable' from left to right or top to bottom. It should show the physiological relevance of the manuscript so readers can assess the importance and content of its findings. Abstract Figures should not merely recapitulate other figures in the manuscript. Please try to keep the diagram as simple as possible and without superfluous information that may distract from the main conclusion(s). Abstract Figures must be provided by authors no later than the revised manuscript stage and should be uploaded as a separate file during online submission labelled as File Type 'Abstract Figure'. Please also ensure that you include the figure legend in the main article file. All Abstract Figures should be created using BioRender. Authors should use The Journal's premium BioRender account to export high-resolution images. Details on how to use and access the premium account are included as part of this email.

EDITOR COMMENTS

Reviewing Editor:

Thank you for submitting your well designed and communicated study. It has generally been well received by our reviewers, but please address their comments/concerns.

REFEREE COMMENTS

Referee #1:

Crew et al present data examining the impact of maternal cardiometabolic dysfunction on offspring vascular reactivity in a novel ovine model of obese pregnancy. This study is well designed and provides comprehensive phenotyping of this model during pregnancy. Well written, I only have a few minor comments.

- For the wire myography analysis how were the subset of ewes determined? Was this a random allocation?

- Given that the study was relatively well powered and the numbers of twins was small in each group, why did the authors include and not just conduct analysis with singletons to reduce any potential variability?

- In figure 8 when separated in to fetal sex there is only 2-7 animals per group, given the low n-number is this an appropriate analysis?

- The authors demonstrate that there is a difference in the placentome characterisation which is suggestive of reduced efficiency in the OB group, why do they not see a reduction in fetal weight?

Referee #2:

This is a well written, interesting manuscript investigating maternal uterine artery adaptation in an obese sheep model. There are some points of clarification that are needed, outlined below.

1. Parity is a factor that changes maternal CV responses to pregnancy and is now an established factor to consider when investigating complicated pregnancy, especially those complicated by obesity. How many previous pregnancies did the ewes have and can the authors account for this in their statistical model.

2. The details of the obesogenic diet appear missing - "ad libitum access to concentrates" What are these concentrates?

3. Were all the pregnancies singleton, did the authors account for sex

4. At what gestational age were the indwelling catheters placed in the ewe

5. A more robust analysis of the placentomes would be to perform a nested Linear mixed effects model where the placentomes are nested within the mother (pregnancy) rather than a 3 way ANOVA, the same could have been done with fetal sex

6. Is the elevation in uterine o2 delivery perhaps associated with increased placental O2 consumption and reduced efficiency? This may also explain why the fetal weight is not different, but the placental weights are increased - particularly the D placentomes...

END OF COMMENTS

Reviewing Editor: *Thank you for submitting your well designed and communicated study. It has generally been well received by our reviewers, but please address their comments/concerns.*

We thank the referees for their insightful comments, which we have addressed below, and in the revised manuscript (with revisions highlighted in yellow). We hope that these changes will make the manuscript suitable for publication in The Journal of Physiology.

Referee #1: *Crew et al present data examining the impact of maternal cardiometabolic dysfunction on offspring vascular reactivity in a novel ovine model of obese pregnancy. This study is well designed and provides comprehensive phenotyping of this model during pregnancy. Well written, I only have a few minor comments.*

1. For the wire myography analysis how were the subset of ewes determined? Was this a random allocation?

Yes, ewes were randomly selected for myography. This has been clarified in the methods (page 11, line 193).

2. Given that the study was relatively well powered and the numbers of twins was small in each group, why did the authors include and not just conduct analysis with singletons to reduce any potential variability?

We have included both twin and singleton pregnancies because of the larger relative number of twins in the instrumented (surgical) group. We have updated the description of these numbers to clarify (page 7, line 114-115). We performed GLMs to assess for the effect of twinning on all biometric and physiological data, and found no significant effect in the instrumented (surgical) or non-instrumented groups (see page 14, line 251-255).

3. In figure 8 when separated into fetal sex there is only 2-7 animals per group, given the low n-number is this an appropriate analysis?

Many thanks for this valid query. The n=2 refers specifically to female OB group in the MCh + LNAME graph (Fig 8G). All other single sex myography groups have n=4-7 animals per group. While this n=2 number is low, there is still a striking sex effect in the MCh + LNAME group overall. Therefore, we believe that it is more informative to present the sexes separately (Fig 8F & 8G), in addition to the combined sex data presented in Fig 8E.

However, we agree with the referee’s concern, so have now raised this as a limitation in the discussion (page 26, line 486-490).

4. The authors demonstrate that there is a difference in the placentome characterisation which is suggestive of reduced efficiency in the OB group, why do they not see a reduction in fetal weight?

Referee 2 suggested an interesting mechanism to explain this pattern (Referee 2, Pt 6), which we have now addressed in the discussion (pg 24, line 438-446).

Referee #2: *This is a well written, interesting manuscript investigating maternal uterine artery adaptation in an obese sheep model. There are some points of clarification that are needed, outlined below.*

1. Parity is a factor that changes maternal CV responses to pregnancy and is now an established factor to consider when investigating complicated pregnancy, especially those complicated by obesity. How many previous pregnancies did the ewes have and can the authors account for this in their statistical model.

Many thanks for raising this interesting point. The ewes were not raised in our facility, so we are unable to provide their specific pregnancy history. However, all ewes are proven breeders of a similar age (2-3 years), and sheep naturally exhibit tightly controlled (seasonal) breeding. Therefore, we can assume that our ewes had 1-2 pregnancies prior to being included in this study. We agree that this is an important consideration, so have now addressed this in the discussion (pg 26, line 490-495).

2. The details of the obesogenic diet appear missing - "ad libitum access to concentrates" What are these concentrates?

Apologies for the confusion - the obese ewes received *ad libitum* access to the same concentrates (Bearts Ewe nuts) as the control ewes. We have edited the description of the feeding regime in the methods to clarify (pg 6, line 92-94).

3. Were all the pregnancies singleton, did the authors account for sex

This study includes both singleton and twin pregnancies. We have updated our description of these numbers for the instrumented (surgical) animals in the methods section (page 7, line 114-115), with the number of twins vs. singletons in the non-instrumented groups described at page 14, lines 257-259. We performed GLMs to account for differences in twinning in both groups, and found none (see page 14, line 251-254).

Similarly, fetal sex was accounted for in all analyses, as indicated at (pg 13, line 247-249, and in each figure legend). Our analyses incorporating fetal sex raised some interesting sex-specific uterine artery adaptations, as outlined in Fig 4, Fig 7 & Fig 8. In

these cases, data were presented as separate fetal sex groups. When no fetal sex effects were present (i.e., all other Figs apart from Fig 4, Fig 7 & Fig 8), the data were presented as a combined group that included both sexes.

4. At what gestational age were the indwelling catheters placed in the ewe

Apologies for the omission, this has been added to the methods (page 7, line 114 & page 9 line 154).

5. A more robust analysis of the placentomes would be to perform a nested Linear mixed effects model where the placentomes are nested within the mother (pregnancy) rather than a 3 way ANOVA, the same could have been done with fetal sex

Many thanks for the suggestion. We initially performed a 3-way ANOVA to account for differences/interactions in diet (CON/OB), fetal sex (male/female) and placental type (A/B/C/D). Because no fetal sex effects were present, male and female data were combined and these data were subsequently assessed for the impact of diet and placentome type with a 2-way ANOVA. We have now repeated this analysis as a mixed effects model, with the data nested within each ewe/mother. Generally, the overall pattern of significance is the same, with some subtle changes to *P* values. These updated *P* values are now presented in Figure 5D-F, and the statistical methods have been edited (page 14, line 250-251) and in the Figure 5 legend to reflect this.

6. Is the elevation in uterine o2 delivery perhaps associated with increased placental O2 consumption and reduced efficiency? This may also explain why the fetal weight is not different, but the placental weights are increased - particularly the D placentomes...

Thank you for this interesting suggestion. We have now incorporated this into the discussion (pg 24, line 438-446).

Dear Dr Crew,

Re: JP-RP-2026-290766R1 "**Maternal cardiometabolic dysfunction and fetal sex-specific alterations to uterine vascular reactivity in an ovine model of obesity during pregnancy**" by Rachael C Crew, Anna LK Cochrane, Youguo Niu, Sage G Ford, Clement L R Cahen, Skaai H Davison, Michael P. Murphy, Susan E Ozanne, and Dino A Giussani

Thank you for submitting your manuscript to The Journal of Physiology. It has been assessed by a Reviewing Editor and by 2 expert referees and we are pleased to tell you that it is acceptable for publication following satisfactory revision.

The referee reports are copied at the end of this email.

Please address all the points raised and incorporate all requested revisions or explain in your Response to Referees why a change has not been made. We hope you will find the comments helpful and that you will be able to return your revised manuscript within 4 weeks. If you require longer than this, please contact journal staff: jp@physoc.org.

Your revised manuscript should be submitted online using the link in your Author Tasks Link Not Available. This link is accessible via your account as Corresponding Author; it is not available to your co-authors. If this presents a problem, please contact journal staff (jp@physoc.org). Image files from the previous version are retained on the system. Please ensure you replace or remove any files that are being revised.

If you do not wish to submit a revised version of your manuscript, you must inform our journal staff (jp@physoc.org) or reply to this email to request withdrawal. Please note that a manuscript must be formally withdrawn from the peer review process at one journal before it may be submitted to another journal.

TRANSPARENT PEER REVIEW POLICY: To improve the transparency of its peer review process The Journal of Physiology publishes online, as supporting information, the peer review history of all articles accepted for publication. Readers will have access to decision letters, including Editors' comments and referee reports, for each version of the manuscript, as well as any author responses to peer review comments. Referees can decide whether or not they wish to be named on the peer review history document.

ABSTRACT FIGURES: Authors are expected to use The Journal's premium BioRender account to create/redraw their Abstract Figures. Information on how to access this account is here: https://physoc.onlinelibrary.wiley.com/journal/14697793/biorender-access.

This will enable Authors to create and download high-resolution figures. If authors have used the free BioRender service, they can use the instructions provided in the link above to download a high-resolution version suitable for publication.

The link provided should only be used for the purposes of this submission. Authors will be charged for figures created on this account if they are not related to this manuscript submission.

LANGUAGE EDITING AND SUPPORT FOR PUBLICATION: If you would like help with English language editing, or other article preparation support, Wiley Editing Services offers expert help, including English Language Editing, as well as translation, manuscript formatting, and figure formatting at www.wileyauthors.com/eeo/preparation. You can also find resources for Preparing Your Article for general guidance about writing and preparing your manuscript at www.wileyauthors.com/eeo/prepresources.

RIGOUR AND REPRODUCIBILITY: We recommend authors consult The Journal's Rigour and Reproducibility requirements to ensure that their manuscript is suitable for publication: https://physoc.onlinelibrary.wiley.com/pb-assets/hub-assets/physoc/documents/TJP-Rigour-and-Reproducibility-Requirements.pdf

REVISION CHECKLIST:

Check that your Methods section conforms to journal policy: https://jp.msubmit.net/cgi-bin/main.plex?form_type=display_requirements#methods

Check that data presented conforms to the statistics policy: https://jp.msubmit.net/cgi-bin/main.plex?form_type=display_requirements#statistics

Upload a full Response to Referees file. To create your 'Response to Referees' copy all the reports, including any comments from the Senior and Reviewing Editors, into a Microsoft Word, or similar, file and respond to each point, using font or background colour to distinguish comments and responses and upload as the required file type.

Please upload two versions of your manuscript text: one with all relevant changes highlighted and one clean version with no changes tracked. The manuscript file should include all tables and figure legends, but each figure/graph should be uploaded as separate, high-resolution files. The journal is now integrated with Wiley's Image Checking service. For further details, see: https://www.wiley.com/en-us/network/publishing/research-publishing/trending-stories/upholding-image-integrity-wileys-

image-screening-service

You may also upload:

- 'Potential Cover Art' for consideration as the issue's cover image
- Appropriate Supporting Information (Video, audio or data set: see https://jp.msubmit.net/cgi-bin/main.plex?form_type=display_requirements#supp).

We look forward to receiving your revised submission.

If you have any queries, please reply to this email and we will be pleased to advise.

Yours sincerely,

Laura Bennet
Senior Editor
The Journal of Physiology

REQUIRED ITEMS

- Papers must comply with the Statistics Policy: https://jp.msubmit.net/cgi-bin/main.plex?form_type=display_requirements#statistics.

In summary:

- If n {less than or equal to} 30, all data points must be plotted in the figure in a way that reveals their range and distribution. A bar graph with data points overlaid, a box and whisker plot or a violin plot (preferably with data points included) are acceptable formats.

- If n > 30, then the entire raw dataset must be made available either as supporting information, or hosted on a not-for-profit repository, e.g. FigShare, with access details provided in the manuscript.

- 'n' clearly defined (e.g. x cells from y slices in z animals) in the Methods. Authors should be mindful of pseudoreplication.

- All relevant 'n' values must be clearly stated in the main text, figures and tables.

- The most appropriate summary statistic (e.g. mean or median and standard deviation) must be used. Standard Error of the Mean (SEM) alone is not permitted.

- Exact p values must be stated. Authors must not use 'greater than' or 'less than'. Exact p values must be stated to three significant figures even when 'no statistical significance' is claimed.

EDITOR COMMENTS

Reviewing Editor:

Comments for Authors to ensure the paper complies with the Statistics Policy (Required):
Please change results to SD or 95% CI from SEM. For benefit of the Journal, I have noted that there are few in text mentions of p values, but the exact values are clearly provided in figures and tables - this is acceptable.

Please read our statistics policy above.

Senior Editor:

Comments for Authors to ensure the paper complies with the Statistics Policy (Required):
The reviewing editor has asked that you consider The Journal's policy regarding the preferred use of SD not SEM. If you can justify why SEM is suitable, then please let us know. Otherwise, can you change your data presentation.

REFEREE COMMENTS

Referee #1:

I am happy with the revisions made by the authors, well done on an interesting well presented study.

Referee #2:

The authors have addressed the reviewer's concerns and questions and the manuscript is improved.

END OF COMMENTS

We thank the referees and Editors for their comments. In order to be able to compare the data in this manuscript to previously published data from our laboratory and other data in this field, which is normally published as mean + SEM, we prefer to maintain our data presented as a mean + SEM, rather than SD. However, as requested, we present all data as individual data points in graphs, and have ensured that all exact P values are shown in all figures and tables. We have adapted Tables 1, 2 and 3 to provide specific P values for non-significant effects (changes highlighted in yellow in the revised manuscript, pages 37-39). We hope that these changes will make the manuscript suitable for publication in The Journal of Physiology.

Reviewing Editor:

Please change results to SD or 95% CI from SEM. For benefit of the Journal, I have noted that there are few in text mentions of p values, but the exact values are clearly provided in figures and tables - this is acceptable.

Senior Editor:

The reviewing editor has asked that you consider The Journal's policy regarding the preferred use of SD not SEM. If you can justify why SEM is suitable, then please let us know. Otherwise, can you change your data presentation.

Referee #1:

I am happy with the revisions made by the authors, well done on an interesting well presented study.

Referee #2:

The authors have addressed the reviewer's concerns and questions and the manuscript is improved.

Dear Dr Crew,

Re: JP-RP-2026-290766R2 "**Maternal cardiometabolic dysfunction and fetal sex-specific alterations to uterine vascular reactivity in an ovine model of obesity during pregnancy**" by Rachael C Crew, Anna LK Cochrane, Youguo Niu, Sage G Ford, Clement L R Cahen, Skaai H Davison, Michael P. Murphy, Susan E Ozanne, and Dino A Giussani

We are pleased to tell you that your paper has been accepted for publication in The Journal of Physiology.

The last Word (or similar) version of the manuscript provided will be used by the Production Editor to prepare your proof. When this is ready you will receive an email containing a link to Wiley's Online Proofing System. The proof should be thoroughly checked and corrected as promptly as possible.

Authors should note that it is too late at this point to offer corrections prior to proofreading. Major corrections at proof stage, such as changes to figures, will be referred to the Editors for approval before they can be incorporated. Only minor changes, such as to style and consistency, should be made at proof stage. Changes that need to be made after proof stage will usually require a formal correction notice.

All queries at proof stage should be sent to: TJP@wiley.com.

Following copyediting and typesetting the accepted version will be published online.

Yours sincerely,

Laura Bennet
Senior Editor
The Journal of Physiology

IMPORTANT POINTS TO NOTE FOLLOWING ACCEPTANCE OF YOUR PAPER:

• IMPORTANT NOTICE ABOUT OPEN ACCESS: To assist authors whose funding agencies mandate immediate public access to published research findings, The Journal of Physiology allows authors to pay an Open Access (OA) fee to have their papers made freely available immediately on publication.

The Corresponding Author will receive an email from Wiley with details on how to register or log in to Wiley Authors where you will be able to place an order.

• You can check if your funder or institution has a Wiley Open Access Account here:
https://authors.wiley.com/author-resources/Journal-Authors/open-access/author-compliance-tool.html

• TRANSPARENT PEER REVIEW POLICY: To improve the transparency of its peer review process, The Journal of Physiology publishes online as supporting information the peer review history of all articles accepted for publication. Readers will have access to decision letters, including Editors' comments and referee reports, for each version of the manuscript as well as any author responses to peer review comments. Referees can decide whether or not they wish to be named on the peer review history document.

• You can help your research get the attention it deserves! Check out Wiley's free Promotion Guide for best-practice recommendations for promoting your work at: www.wileyauthors.com/eeo/guide. You can learn more about Wiley Editing Services which offers professional video, design, and writing services to create shareable video abstracts, infographics, conference posters, lay summaries, and research news stories for your research at: www.wileyauthors.com/eeo/promotion.

• If you would like to receive our 'Research Roundup', a monthly newsletter highlighting the cutting-edge research published in The Physiological Society's family of journals (The Journal of Physiology, Experimental Physiology, Physiological Reports, The Journal of Nutritional Physiology and The Journal of Precision Medicine: Health and Disease), please click this link, fill in your name and email address and select 'Research Roundup':
https://www.physoc.org/journals-and-media/membernews

EDITOR COMMENTS

Reviewing Editor:

Thank you for your amendments. Congratulations on an excellent study.

Senior Editor:

Comments for Authors to ensure the paper complies with the Statistics Policy:
They are using SEM, but this is consistent with previous data pools and it does not materially change the information

----
